# Sleep disorder is associated with increased risk of major adverse cardiovascular events in patients with schizophrenia

**DOI:** 10.3389/fneur.2025.1601319

**Published:** 2025-07-25

**Authors:** Jinbo Wu, Tingting Wang, Xiaomei Jiang

**Affiliations:** ^1^Department of Psychiatry, The First Affiliated Hospital of Harbin Medical University, Harbin, China; ^2^Department of Endocrinology, Harbin Red Cross Central Hospital, Harbin, China

**Keywords:** sleep disorder, sleep duration, insomnia, daytime sleepiness, MACE, schizophrenia

## Abstract

**Objective:**

To evaluate the association of sleep disorder with the risk of major adverse cardiovascular events (MACEs) among patients with schizophrenia—a population known to have heightened cardiometabolic vulnerability, yet underexplored in terms of sleep-related cardiovascular risk.

**Methods:**

The cross-sectional study included 1,072 participants diagnosed with schizophrenia between January and December 2022. The sleep disorder was defined based on self-reported sleep duration, insomnia, and daytime sleepiness, and collected via self-completed questionnaire. Patients’ MACEs including fatal and non-fatal myocardial infarction (MI), fatal and non-fatal stroke, and cardiovascular death were collected from chart review. Multivariate logistic regression model was employed to assess the association of sleep disorders with the risk of MACE after controlling for potential confounding factors.

**Results:**

Sleep disorders were common, with 25.7% reporting insomnia, 30.0% reporting short sleep duration (<6 h), and 36.0% experiencing excessive daytime sleepiness. Among the 1,072 patients with schizophrenia, 20.3% experienced a MACE. Participants who have insomnia, short duration of sleep or excessive daytime sleepiness were more likely to have MACEs compared with those without these sleep disorders (all *p* < 0.01). Multivariate logistic regression indicated that insomnia (OR = 1.88, 95% CI: 1.26–2.78; *p* < 0.01), short sleep duration (OR = 1.66, 95% CI: 1.17–2. 35; *p* < 0.01), and excessive daytime sleepiness (OR = 1.55, 95% CI: 1.13–2.12; *p* < 0.01) were significantly associated with the risk of MACE after controlling for potential confounding factors.

**Conclusion:**

Sleep disorders are significantly associated with a higher risk of MACEs in patients with schizophrenia.

## Introduction

Schizophrenia affects approximately 24 million people worldwide, with a lifetime prevalence of about 1% (1). Beyond the profound impact on mental health, individuals with schizophrenia face a markedly increased risk of cardiovascular diseases (CVDs)—the leading cause of mortality in this population (2, 3). Epidemiological studies suggest that people with schizophrenia have a two-to threefold higher risk of CVDs compared to the general population, leading to a reduction in life expectancy by an average of 10–20 years (4, 5). This elevated risk is thought to stem from a combination of lifestyle factors, metabolic side effects of antipsychotic medications, and underlying neurobiological changes (2, 6).

Sleep disorders are also highly prevalent in schizophrenia, with rates of insomnia and excessive daytime sleepiness reported to affect up to 80% of patients (7). In the general population, sleep disturbances—particularly insomnia, short sleep duration, and excessive daytime sleepiness—have been independently linked to higher cardiovascular risk, driven by mechanisms such as autonomic dysfunction, systemic inflammation, and metabolic disturbances (8, 9). These pathways may be further intensified in individuals with schizophrenia due to disorder-specific characteristics. Second-generation antipsychotics, for example, are known to cause metabolic side effects including weight gain, insulin resistance, and dyslipidemia, all of which contribute to cardiovascular burden (10). Additionally, schizophrenia is frequently associated with dysregulation of the hypothalamic–pituitary–adrenal (HPA) axis, leading to elevated cortisol levels, increased sympathetic nervous system activity, and chronic inflammation (11). These factors may amplify the detrimental cardiovascular effects of poor sleep, including hypertension, endothelial dysfunction, and atherosclerosis, resulting in a heightened risk of major adverse cardiovascular events (MACEs).

This study aims to investigate the association between specific sleep disorders—insomnia, short sleep duration, and daytime sleepiness—and the incidence of MACEs in patients with schizophrenia. While sleep disturbances have been linked to cardiovascular disease in the general population, their impact on cardiovascular outcomes in individuals with schizophrenia remains largely understudied. By focusing on this unique clinical subgroup, we aim to fill a crucial gap in the literature and assess whether sleep management could be a viable strategy to mitigate cardiovascular risk in this particularly vulnerable population.

## Materials and methods

### Study design and population

This cross-sectional study included consecutive patients diagnosed with schizophrenia who were treated at The First Affiliated Hospital of Harbin Medical University between January and December 2022. Eligible participants were adults (≥18 years) with a documented diagnosis of schizophrenia based on Diagnostic and Statistical Manual of Mental Disorders-5 (DSM-5) criteria (12). We excluded individuals with cognitive impairments that prevented informed consent or those with incomplete medical records. A total of 1,072 patients met the inclusion criteria and were enrolled in the study after providing informed consent. This study was approved by the Institutional Review Board of The First Affiliated Hospital of Harbin Medical University to ensure ethical compliance.

### Sleep disorder assessment

Sleep disorders were evaluated through a structured self-administered questionnaire, adapted from validated sleep assessment tools. Three specific dimensions of sleep were measured:

- Insomnia: Assessed based on self-reported difficulty in falling asleep (prolonged sleep latency ≥30 min), maintaining sleep (nocturnal awakenings with inability to return to sleep), or early-morning awakenings (waking ≥30 min before desired time without returning to sleep) (13).
- Sleep Duration: Defined as the average sleep duration in every 24 h and categorized into short (<6 h), normal (6–8 h), and long (>8 h) sleep duration (14).
- Daytime Sleepiness: Measured using the Epworth Sleepiness Scale (15), an 8-item validated tool assessing likelihood of dozing in daily activities (e.g., watching TV, driving). Items were scored 0–3 (total range: 0–24), with scores >10 indicating excessive daytime sleepiness. This scale has been validated for use in schizophrenia populations.

### Data collection and outcomes

The primary outcome was the incidence of MACEs, defined as fatal or non-fatal myocardial infarction (MI), fatal or non-fatal stroke, and cardiovascular death. MACE data were collected from patient medical records and confirmed by two independent cardiologists to ensure accurate classification.

Several covariates were included to control for confounding variables, based on their known associations with CVD and sleep disorders. These covariates included demographic variables (age, gender, body mass index [BMI]), smoking status, marital status, employment status, comorbidities (presence of hypertension, diabetes mellitus, and hyperlipidemia) and antipsychotic medications. Although marital and employment status are not direct biological risk factors for cardiovascular disease, they were included as covariates due to their established associations with health behaviors, stress exposure, healthcare access, and treatment adherence. Prior research in both general and psychiatric populations suggests that social determinants such as social isolation, unemployment, or lack of spousal support can independently influence cardiovascular outcomes. Therefore, we included these variables to better control for potential psychosocial confounding.

### Statistical analysis

All analyses were conducted using SPSS version 22.0. Descriptive statistics summarized baseline characteristics. The association between sleep disorders and MACE was examined using multivariate logistic regression models. Initially, unadjusted odds ratios (ORs) were calculated for each sleep disorder. Multivariate models were then constructed, adjusting for potential confounders, including age, gender, BMI, smoking, employment status, hypertension, diabetes, and hyperlipidemia. The associations were presented as adjusted ORs with 95% confidence intervals (CIs). Statistical significance was set at *p* < 0.05 for all analyses.

Missing data on key variables, including sleep measures and cardiovascular outcomes, were addressed using multiple imputations by chained equations (MICE). This approach assumed data were missing at random (MAR) and aimed to reduce bias and maintain statistical power.

## Results

A total of 1,072 patients with schizophrenia were included in the study, with a mean age of 52.2 years. Of these, 53.5% were male, 26.9% were current smokers, and 35.0% had normal BMI. Among the participants, 17.9% had a history of hypertension, 14.6% had diabetes mellitus, and 22.6% had hyperlipidemia. Regarding antipsychotic use, 82.7% were prescribed second-generation agents only, 9.2% first-generation only, and 8.0% a combination of both. Compared to those without MACEs, patients with MACEs were older, more likely to be obese or current smokers, and had higher rates of hypertension and diabetes (all *p* < 0.05). Significant group differences were also observed in antipsychotic drug patterns (*p* = 0.03), with a lower proportion of first-generation antipsychotic use among MACE patients. The demographic and clinical profiles of the participants are summarized in Table 1.

**Table 1:** Sociodemographic and clinical characteristics of patients with schizophrenia.

Variables	Total	Patients with MACEs	Patients without MACEs	*p* value
N	1,072	218	854	
Age, years	52.2 ± 6.0	53.5 ± 6.0	51.8 ± 5.9	<0.01
Gender, n (%)				0.79
Male	574 (53.5%)	115 (52.8%)	459 (53.7%)	
Female	498 (46.5%)	103 (47.2%)	395 (46.3%)	
Body mass index, n (%)				<0.01
Underweight (<18.5 kg/m^2^)	35 (3.3%)	5 (2.3%)	30 (3.5%)	
Normal (18.5–23.9 kg/m^2^)	375 (35.0%)	71 (32.6%)	304 (35.6%)	
Overweight (24–27.9 kg/m^2^)	486 (45.3%)	69 (31.7%)	417 (48.8%)	
Obesity (≥28 kg/m^2^)	176 (16.4%)	73 (33.5%)	103 (12.1%)	
Smoking, n (%)				0.02
Never smoker	472 (44.0%)	87 (39.9%)	385 (45.1%)	
Ex-smoker	312 (29.1%)	56 (25.7%)	256 (30.0%)	
Current smoker	288 (26.9%)	75 (34.4%)	213 (24.9%)	
Marital status				0.15
Married	265 (24.7%)	62 (28.4%)	156 (71.6%)	
Others	807 (75.3%)	203 (23.8%)	651 (76.2%)	
Employment, n (%)				0.35
Unemployed	463 (43.2%)	88 (40.4%)	130 (59.6%)	
Employed or retired	609 (56.8%)	375 (43.9%)	479 (56.1%)	
Hypertension, n (%)				0.02
No	880 (82.1%)	167 (76.6%)	710 (83.1%)	
Yes	192 (17.9%)	51 (23.4%)	144 (16.9%)	
Diabetes mellitus, n (%)				<0.01
No	915 (85.4%)	169 (77.5%)	746 (87.4%)	
Yes	157 (14.6%)	49 (22.5%)	108 (12.6%)	
Hyperlipidemia, n (%)				0.16
No	830 (77.4%)	161 (73.9%)	669 (78.3%)	
Yes	242 (22.6%)	57 (26.1%)	185 (21.7%)	
Antipsychotic drugs, n (%)				0.03
1^st^ generation only	99 (9.2%)	10 (4.6%)	89 (10.4%)	
2^nd^ generation only	887 (82.7%)	189 (86.7%)	698 (81.7%)	
Both 1^st^ and 2^nd^ generations	86 (8.0%)	19 (8.7%)	67 (7.8%)	

**Figure 1:**
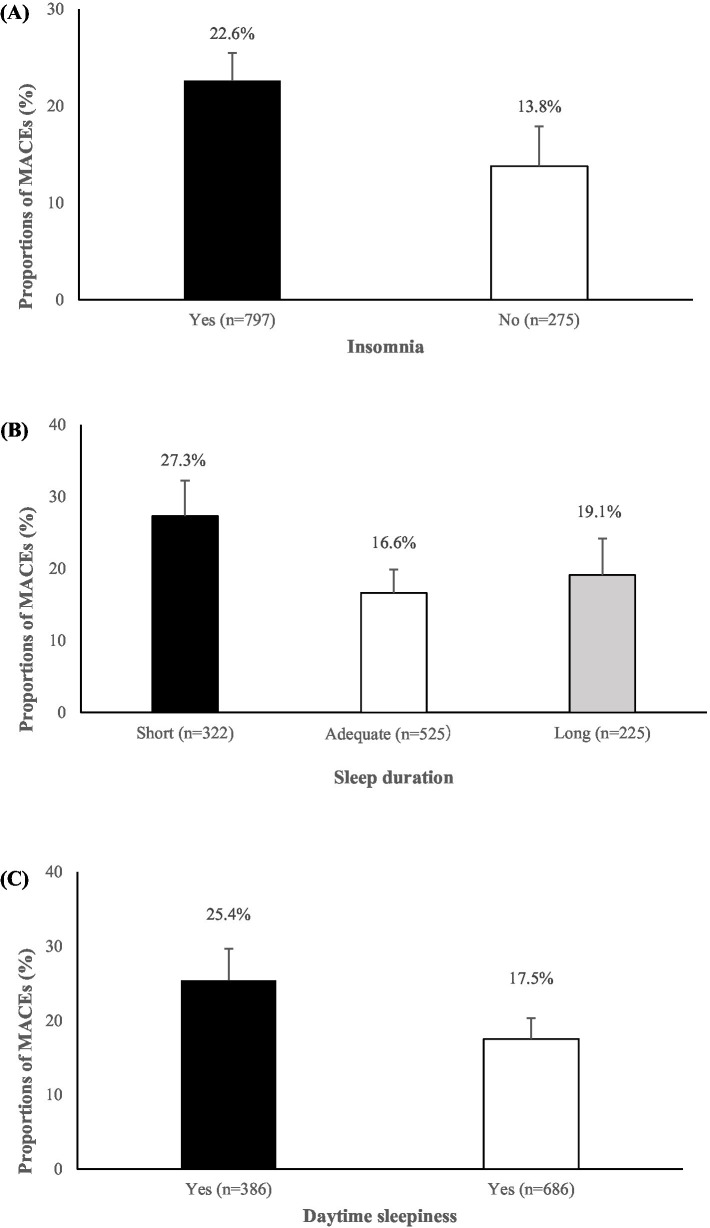
Proportions of major adverse cardiovascular events (MACEs) among schizophrenia patients categorized by the presence or absence of insomnia **(A)**, varying sleep durations **(B)**, and the presence or absence of excessive daytime sleepiness **(C)**. Group differences were assessed using chi-square tests; all comparisons were statistically significant (*p* < 0.01).

The prevalence of sleep disorders was high among the study participants, with 25.7% reporting insomnia, 30.0% reporting short sleep duration (<6 h per night), and 36.0% experiencing excessive daytime sleepiness (Epworth Sleepiness Scale score >10). Among the study population, 20.3% (*n* = 218) experienced a MACE. As shown in Figure 1, participants who have insomnia, short duration of sleep or excessive daytime sleepiness were more likely to have MACEs compared with those without these sleep disorders (all *p* < 0.01).

As shown in Table 2, in the unadjusted analysis, each of the three sleep disorders—insomnia, short sleep duration, and excessive daytime sleepiness—was significantly associated with an increased risk of MACE. Patients with insomnia had an unadjusted OR of 1.82 (95% CI: 1.24–2.66, *p* < 0.01), those with short sleep duration had an OR of 1.89 (95% CI: 1.35–2.64, *p* < 0.01), and those with daytime sleepiness had an OR of 1.61 (95% CI: 1.19–2.17, *p* < 0.01). In multivariate logistic regression analysis, insomnia (OR = 1.88, 95% CI: 1.26–2.78; *p* < 0.01), short sleep duration (OR = 1.66, 95% CI: 1.17–2. 35; *p* < 0.01), and excessive daytime sleepiness (OR = 1.55, 95% CI: 1.13–2.12; *p* < 0.01) remained significantly associated with an increased risk of MACE, after adjusting for age, gender, BMI, smoking status, marital status, employment status, presence of hypertension, diabetes mellitus, hyperlipidemia, and antipsychotic drug use. These associations were statistically significant, suggesting that each of these sleep disorders independently contribute to the risk of adverse cardiovascular outcomes in patients with schizophrenia.

**Table 2:** Multivariate logistic regression analysis to explore the association of sleep disorders with the risk of major adverse cardiovascular events (MACEs).

Variables	Univariate analysis			Multivariate analysis*		
	OR	95% CI	*P* value	OR	95% CI	*P* value
Insomnia						
No	Ref.			Ref.		
Yes	1.82	1.24–2.66	<0.01	1.88	1.26–2.78	<0.01
Sleep duration						
Adequate	Ref.			Ref.		
Long	1.20	0.80–1.79	0.39	1.24	0.82–1.88	0.31
Short	1.89	1.35–2.64	<0.01	1.66	1.17–2.35	<0.01
Daytime sleepiness						
No	Ref.			Ref.		
Yes	1.61	1.19–2.17	<0.01	1.55	1.13–2.12	<0.01

* In the multivariate logistic regression model, potential confounding factors include age, gender, BMI, smoking status, marital status, employment status, presence of hypertension, diabetes mellitus, hyperlipidemia, and antipsychotic drugs.

## Discussion

### Principal findings

This study demonstrates a significant association between sleep disorders and the risk of MACE in patients with schizophrenia. Specifically, insomnia, short sleep duration, and excessive daytime sleepiness were each independently associated with increased odds of MACE, even after adjusting for traditional cardiovascular risk factors such as age, hypertension, diabetes, and hyperlipidemia. These findings highlight the importance of sleep disturbances as potential risk factors for cardiovascular morbidity in schizophrenia and suggest that addressing sleep disorders may play a role in reducing cardiovascular risks in this vulnerable population.

### Comparison with prior research

Our results align with previous studies that have linked sleep disorders to cardiovascular disease risk in the general population. Large cohort studies have shown that inadequate sleep duration, particularly less than 6 h per night, is associated with increased risks of cardiac mortality (16), while insomnia and excessive daytime sleepiness have also been implicated as cardiovascular risk factors (17).

In patients with schizophrenia, these risks may be further amplified due to unique biological characteristics and medication effects. Many patients with schizophrenia experience chronic physiological stress, heightened sympathetic nervous system activity, and persistent inflammation, all of which can compound the cardiovascular effects of sleep disturbances (18). Furthermore, antipsychotic medications—particularly second-generation antipsychotics—are associated with significant metabolic side effects, including weight gain, insulin resistance, and dyslipidemia, which elevate cardiovascular risk (19). Some antipsychotics also influence sleep architecture, leading to either sedation or fragmented sleep, thereby exacerbating sleep-related circadian disruption (20). In our study, we further accounted for the effects of antipsychotic medication by including drug class (first-generation, second-generation, or both) in the multivariate analysis. The association between sleep disorders and MACE remained statistically significant even after this adjustment, suggesting that sleep disturbances confer independent cardiovascular risk beyond the influence of antipsychotic class. However, due to limitations in data granularity, we were unable to evaluate the impact of specific drugs or dosages. Future studies with detailed pharmacological profiles are needed to explore drug-specific cardiovascular effects.

Consistent with our findings, large-scale data by Berry et al. from the UK Biobank also supports the link between sleep disturbance and cardiovascular risk in schizophrenia. In that study, patients with schizophrenia who reported insomnia (PR = 1.37, 95% CI: 1.02–1.85; *p* = 0.04), excessive daytime sleepiness (PR = 1.35, 95% CI: 1.09–1.66; *p* = 0.01), or short sleep duration (PR = 1.32, 95% CI: 1.03–1.71; *p* = 0.03) had significantly higher MACE prevalence compared with those without such symptoms (21). Notably, their reported prevalence ratios (1.32–1.37) were slightly lower than our adjusted odds ratios (1.55–1.88), which may reflect differences in sample characteristics, outcome ascertainment, or statistical approach.

### Biological mechanisms linking sleep disorders to cardiovascular risk

Several mechanisms may explain the association between sleep disorders and cardiovascular events in schizophrenia. Disrupted sleep is known to increase sympathetic nervous system activity, elevate blood pressure, and trigger the HPA axis, leading to higher levels of circulating stress hormones such as cortisol (22). Over time, these physiological changes can result in endothelial dysfunction, arterial stiffness, and increased atherosclerotic burden, all of which are key contributors to cardiovascular events (23).

In patients with schizophrenia, HPA axis dysregulation may be particularly pronounced, possibly due to chronic stress exposure and impaired feedback inhibition (24). Prior studies have demonstrated that this population tends to exhibit altered cortisol awakening responses, prolonged stress hormone secretion, and impaired negative feedback regulation of the HPA axis (25, 26). These abnormalities may lead to sustained hypercortisolemia, which has been shown to induce visceral fat accumulation, insulin resistance, and systemic inflammation—each of which independently increases cardiovascular risk. Furthermore, chronic activation of the HPA axis may exacerbate sleep fragmentation and circadian misalignment, creating a vicious cycle that perpetuates both sleep and cardiometabolic disturbances. These abnormalities may magnify the cardiovascular impact of sleep disruption, making the schizophrenia population more vulnerable to MACE when exposed to chronic sleep disturbances.

In addition to HPA axis dysregulation, several schizophrenia-specific biological pathways may contribute to heightened cardiovascular risk when sleep disorders are present. Chronic low-grade systemic inflammation is frequently observed in patients with schizophrenia, with elevated levels of inflammatory cytokines such as interleukin-6 (IL-6), tumor necrosis factor-alpha (TNF-*α*), and C-reactive protein (CRP). These inflammatory markers are independently associated with increased cardiovascular risk and are also known to be exacerbated by sleep disruption. Moreover, autonomic nervous system (ANS) dysfunction—manifested as elevated sympathetic tone and reduced heart rate variability—is another common feature in schizophrenia and is similarly aggravated by poor sleep. This autonomic imbalance may predispose patients to arrhythmias, hypertension, and endothelial dysfunction. Oxidative stress and mitochondrial dysfunction, both implicated in schizophrenia pathophysiology, may also interact with sleep abnormalities to accelerate vascular injury. Together, these factors suggest that schizophrenia-specific vulnerabilities amplify the deleterious cardiovascular effects of sleep disorders.

Notably, excessive daytime sleepiness may serve as a clinical indicator of these underlying neuroendocrine and metabolic disturbances. Excessive daytime sleepiness is a frequent side effect of antipsychotic medications—particularly second-generation agents with strong sedative or antihistaminergic properties—which can cause residual somnolence during the day. However, schizophrenia itself is associated with circadian rhythm dysregulation, including blunted melatonin rhythms, phase delays, and disrupted sleep–wake patterns. These intrinsic abnormalities may contribute to excessive daytime sleepiness independently of medication use. In our analysis, the association between excessive daytime sleepiness and MACE persisted even after adjusting for antipsychotic class, suggesting that excessive daytime sleepiness may reflect an underlying pathophysiological state that predisposes individuals to cardiovascular risk, potentially through mechanisms such as autonomic imbalance or metabolic dysfunction. In addition, sleep disturbances may further worsen metabolic health, as poor sleep is linked to insulin resistance, dyslipidemia, and increased visceral fat—all conditions commonly observed in schizophrenia patients, partly due to antipsychotic medications (27).

Taken together, the synergistic effects of disrupted sleep, heightened HPA axis activation, and schizophrenia-specific vulnerabilities (including medication-related metabolic side effects) synergistically contribute to heightened cardiovascular risk. Our study supports the hypothesis that sleep disorders could act as independent contributors to cardiovascular risk, suggesting that sleep management could be a modifiable target for cardiovascular risk reduction in this high-risk population.

### Clinical implications and intervention opportunities

The clinical implications of these findings are significant. Routine screening for sleep disorders in patients with schizophrenia may facilitate early identification and intervention for cardiovascular risk. Specific interventions aimed at improving sleep quality—such as cognitive behavioral therapy for insomnia (CBT-I) and pharmacological treatments like melatonin for insomnia—should be considered alongside lifestyle modifications. Recent studies have also shown promising effects of adjunctive sleep interventions such as bright light therapy and sleep hygiene education in improving sleep quality and reducing symptom severity in schizophrenia (28, 29). These non-pharmacological approaches may offer additional benefits for cardiovascular health by normalizing circadian rhythms and improving overall sleep architecture. Additionally, careful monitoring of sleep patterns is essential when prescribing antipsychotic medications, as some agents have pronounced sedative effects that may prolong sleep duration but disrupt sleep architecture and circadian rhythms, potentially increasing cardiovascular risk.

It is worth noting that the sample size of the long sleep duration group was smaller than that of other sleep duration categories, which may limit the statistical power to detect associations in this subgroup. Moreover, the lack of a significant association between long sleep duration and cardiovascular events may partly reflect the sedative effects of antipsychotic medications, which can prolong sleep duration without improving sleep quality. Medication-induced extended sleep might involve disrupted sleep architecture or circadian rhythm alterations, potentially masking true cardiovascular risk related to long sleep. Future studies incorporating objective sleep measurements and detailed pharmacological data are needed to clarify these complex interactions. Therefore, the associations observed for long sleep should be interpreted with caution, and future studies with larger sample sizes are warranted to confirm these findings.

### Strengths and limitations

This study has several strengths, including a well-defined sample of patients with schizophrenia and comprehensive control for cardiovascular risk factors.

However, there are limitations to consider. First and foremost, the cross-sectional design of this study precludes any causal inference regarding the relationship between sleep disorders and cardiovascular events. As such, the observed associations do not establish temporal or causal links. To address this critical limitation, future longitudinal cohort studies are necessary to clarify the directionality and causality of these relationships, as well as to evaluate whether interventions targeting sleep disturbances can reduce cardiovascular risk in patients with schizophrenia.

Second, sleep disorders were assessed using self-reported questionnaires, which may introduce recall bias and reporting inaccuracies, potentially limiting the objectivity and validity of the findings. To improve the accuracy and reliability of sleep assessments, future studies should incorporate objective measurements such as actigraphy or polysomnography.

Finally, despite adjustment for several confounders, potential residual confounding by unmeasured factors—such as physical activity levels, dietary habits, and medication adherence—may still influence the observed associations. These factors should be carefully considered and measured in future research to better isolate the effects of sleep disorders on cardiovascular risk.

## Conclusion

In conclusion, our findings indicate that sleep disorders are significantly associated with a higher risk of MACEs in patients with schizophrenia. Addressing sleep disturbances may represent an actionable intervention to mitigate cardiovascular risk in this population. Further research is needed to evaluate the long-term impact of sleep disorder treatment on cardiovascular outcomes and to develop targeted interventions that address the unique needs of patients with schizophrenia.

## Data Availability

The raw data supporting the conclusions of this article will be made available by the authors, without undue reservation.
